# Survival outcomes and prognostic value of nutritional and inflammatory markers in third-line treatment of metastatic pancreatic cancer

**DOI:** 10.3389/fonc.2026.1713376

**Published:** 2026-03-24

**Authors:** Júlia Vidulin, Dalibor Ondruš, Śtefan Pöršök, Melinda Kráčalíková, Vanda Ušáková, Kristián Šuták, Michaela Miškovičová, Marína Kizeková, Maroš Fremal, Jana Pisáková, Ivan Kecskés, Jaroslava Lešková, Branislav Bystrický

**Affiliations:** 1 Department of Oncology, Faculty Hospital, Trenčín, Slovakia; 2 1st Department of Oncology, Faculty of Medicine, Comenius University in Bratislava and St. Elisabeth Cancer Institute, Bratislava, Slovakia; 3 2nd Department of Oncology, Faculty of Medicine, Comenius University in Bratislava and National Cancer Institute, Bratislava, Slovakia; 4 Department of Oncology, Faculty Hospital, Trnava, Slovakia; 5 Department of Oncology, Faculty Hospital, Nitra, Slovakia; 6 Department of Oncology, F. D. Roosevelt University Hospital, Banská Bystrica, Slovakia; 7 Department of Oncology, Merciful Brothers Hospital, Bratislava, Slovakia; 8 Department of Radiotherapy and Oncology, Faculty of Medicine, PJ Safarik University and Eastern Slovakia Institute of Oncology, Košice, Slovakia; 9 Oncology Centre, University Hospital, Martin, Slovakia; 10 Department of Medical Oncology, Faculty Hospital, Prešov, Slovakia; 11 Faculty of Healthcare, Alexander Dubček University of Trenčín, Trenčín, Slovakia

**Keywords:** C-reactive protein (CRP), glasgow prognostic score (GPS), inflammatory biomarkers, metastatic pancreatic cancer, neutrophil-to-lymphocyte ratio (NLR), nutritional biomarkers, prognostic nutritional index (PNI), systemic inflammation response index (SIRI)

## Abstract

**Background:**

Metastatic pancreatic ductal adenocarcinoma (mPDAC) is one of the most aggressive malignancies with a very poor prognosis. Currently, there is no standardized therapeutic approach for third-line systemic treatment. The aim of this retrospective/prospective study was to evaluate survival outcomes in patients treated in the third-line setting and to analyze the impact of nutritional and inflammatory biomarkers on overall survival (OS) and progression-free survival (PFS).

**Methods:**

The analysis retrospectively and prospectively included 95 patients with mPDAC treated with third-line systemic therapy. Data were collected from the Slovak Pancreas Registry. Data were collected from 13 hospitals. Baseline chemotherapy regimens, ECOG, nutritional, and inflammatory parameters were recorded and used to calculate prognostic scores (PNI, CRP, NLR, SII, SIRI, GPS). Their association with OS and PFS was analyzed using the log-rank test and Kaplan-Meier survival analysis.

**Results:**

The most frequently used regimen was FOLFOX/CAPOX (56 patients), followed by nanoliposomal irinotecan (nal-IRI, 24 patients) and capecitabine (11 patients). The median OS was 5.55 months and the median PFS was 3.29 months. A PNI > 40.5 was significantly associated with longer OS (6.34 vs. 3.32 months, *p* = 0.001) and PFS (3.71 vs. 2.76 months, *p* = 0.009). Lower GPS scores were significantly associated with improved OS (*p* = 0.006) and PFS (*p* = 0.027). Other inflammatory markers (NLR, SIRI, SII, CRP) did not show a statistically significant impact on OS or PFS.

**Conclusion:**

Third-line systemic therapy may offer clinical benefit in selected mPDAC patients, particularly in those with favorable nutritional-inflammatory profiles. The Prognostic Nutritional Index (PNI) and Glasgow Prognostic Score (GPS) appear to be potentially useful tools for identifying candidates suitable for further systemic treatment. However, further prospective studies are needed to confirm these findings.

## Introduction

Metastatic pancreatic ductal adenocarcinoma (mPDAC) is a malignant disease occurring mainly in developed countries with one of the lowest survival rates among solid tumors (1). Most patients are diagnosed at an inoperable or metastatic stage of the disease (2). Despite advances in first- and second-line treatment, the prognosis for patients with mPDAC remains poor. Most patients progress despite these therapies, making third-line treatment an increasingly relevant topic (2, 3). Currently, there are no clear guidelines for treatment following second-line failure. In clinical practice, chemotherapy remains the most common choice in the third line setting for mPDAC. Only a few agents are available for use in this context, and many clinicians decide for re-challenge strategies. Immunotherapy and targeted therapies are also gaining attention (4).

Between 2013 and 2023, Lu et al. were the first to compare 72 patients following second-line chemotherapy failure. The greatest benefit was seen with a combination of chemotherapy and immunotherapy (median progression-free survival [mPFS] 5.2months, median overall survival [mOS] 5.9months, disease control rate [DCR] 31.3%), although at the cost of increased toxicity. A statistically significant difference was observed between the symptomatic/palliative care group and the treated group (mOS, *p* < 0.001; mPFS, *p* < 0.001; DCR, *p* < 0.001) (2). Dean et al. studied the use of gemcitabine + nab-paclitaxel in third-line treatment in patients with ECOG PS (performance status according to Easter Cooperative Oncology Group) 0, previously treated with this regimen in the first line and subsequently with FOLFIRINOX in the second line, with promising results (5). Promising efficacy and safety have also been reported for the combination of calmodulin-binding peptide, cisplatin, and nivolumab (6). The most recent study from 2025 by Evrard et al. included 202 patients with advanced PDAC; the most commonly used regimen was FOLFIRI (25.2%), median PFS was 2.2 months, and median OS was 4.2 months (7).

### Nutritional and inflammatory biomarkers

Several factors influence treatment response and overall survival, including nutritional and inflammatory biomarkers. Nutritional markers include obesity, body mass index (BMI), serum albumin levels, performance status, and the Prognostic Nutritional Index (PNI) (6). The ECOG PS and Karnofsky performance scale are commonly used in clinical practice to assess a patient’s functional capacity. ECOG is a five-point scale that evaluates a patient’s ability to perform daily activities and the level of need of assistance, where higher scores are associated with poorer prognosis and treatment response (8, 9).

The PNI is a simple formula based on serum albumin and lymphocyte levels, reflecting both nutritional and inflammatory status. There is no universally accepted cut-off value, but studies most frequently use values of 40.5 or 45, with lower values indicating a poorer prognosis (10–13).

Inflammatory markers include the Glasgow Prognostic Score (GPS), calculated from serum albumin and C-reactive protein (CRP) levels. A score of 0 corresponds to serum albumin > 35 g/L and CRP < 10 mg/L; a score of 1 corresponds to either CRP > 10 mg/L with normal albumin or albumin < 35 g/L with normal CRP; a score of 2 corresponds to CRP > 10 mg/L and albumin < 35 g/L. Higher scores are associated with worse prognosis (6, 14).

CRP alone may also serve as an individual marker, with elevated levels correlating with worse outcomes. A cut-off value of 10 mg/L is commonly used (15, 16). Another potential marker is the neutrophil-to-lymphocyte ratio (NLR). Normal values range from 1–2, while values > 3.0 or < 0.7 are considered abnormal. A “gray zone” of 2.3–3.0 may indicate early pathologic changes. Commonly used cut-off values are 2.5 and 5 (17–20).

The Systemic Inflammation Response Index (SIRI) is calculated from absolute neutrophil, monocyte, and lymphocyte counts. Higher values indicate a poorer prognosis, with a commonly used cut-off of 2.3 (21, 22). The Systemic Immune-Inflammation Index (SII) is calculated from platelet and neutrophil counts relative to lymphocyte counts. Higher values are associated with worse outcomes, although no consensus on the exact cut-off has been reached. A value of SII > 900 has shown promising results (23, 24).

### Objective

The aim of this study is to analyze outcomes of patients treated with third-line systemic therapy for metastatic pancreatic adenocarcinoma, assess the impact of nutritional and inflammatory markers on overall survival and progression-free survival, and contribute to a better understanding of this clinically relevant but underexplored area.

## Methods

A retrospective/prospective analysis was performed on 95 patients with mPDAC who were treated with 3^rd^ line chemotherapy. Data were collected within the Slovak Pancreatic Register (SlovPac), national multicenter registry and included 13 centers. The cohort included 47 women and 48 men. All diagnoses were histologically confirmed, and third-line therapy was initiated after second-line treatment failure.

Patients were included if complete information on third-line systemic therapy and survival outcomes was available. As data were collected retrospectively from a national registry, detailed baseline data on patient comorbidities and frailty were not systematically recorded across participating centers and were therefore unavailable for uniform analysis.

**Figure 1:**
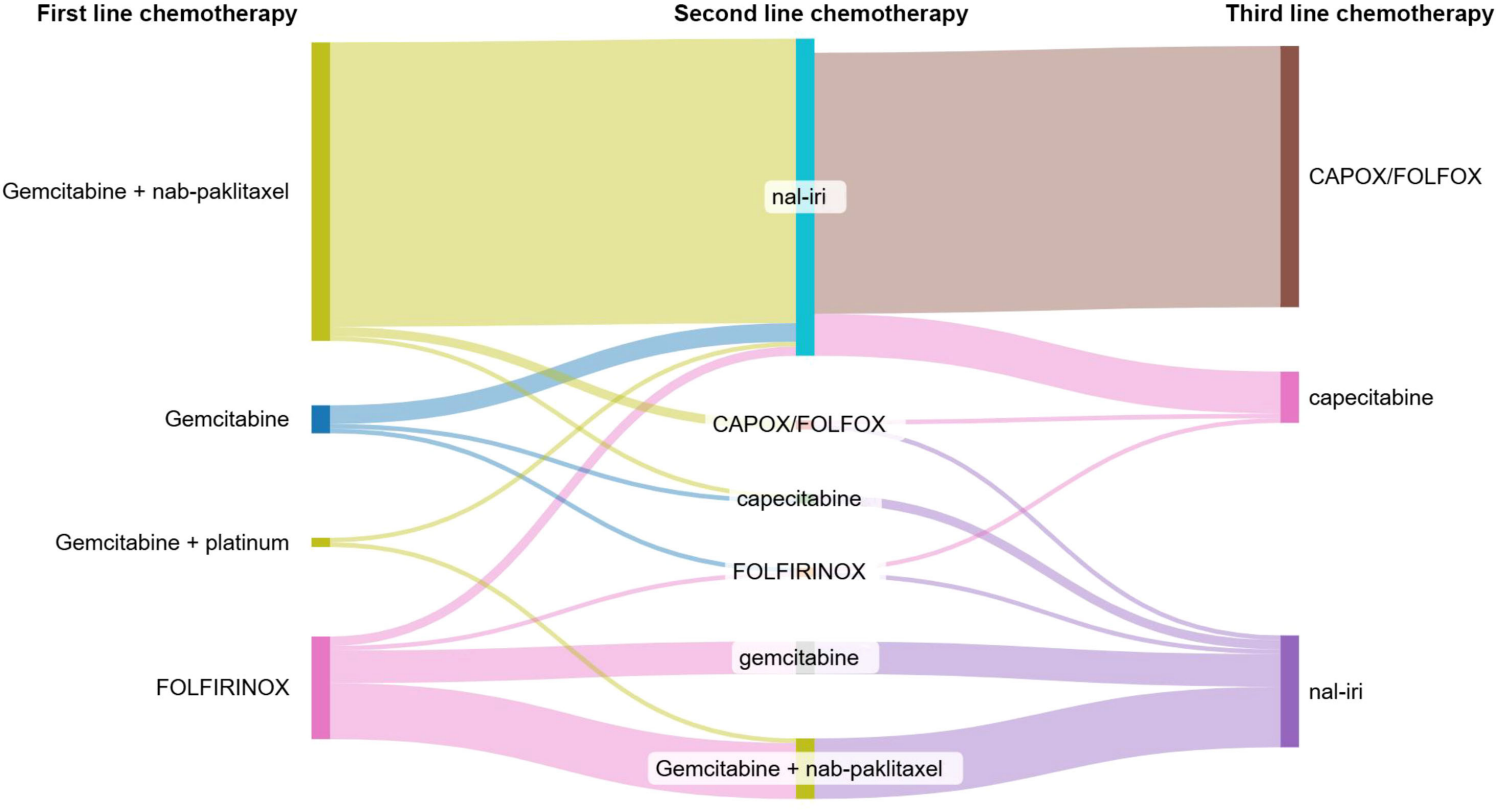
Overview of first-, second-, and third-line chemotherapy regimens in mPDAC.

Eligibility for third-line therapy was determined by the treating physician, who assessed the patient’s overall condition and age and evaluated whether the treatment was expected to provide a benefit in terms of quality of life. The choice of treatment regimen depended on the previously administered therapy; a complete overview of treatment regimens up to third-line therapy is presented in **Figure 1**.

Disease progression or treatment failure was primarily assessed by computed tomography according to RECIST version 1.1. In cases where radiological assessment was not feasible, disease progression could also be determined based on an increase in serum CA19–9 levels, clinical deterioration, or a decline in performance status resulting in inability to continue treatment. In such cases, progression was determined by the treating physician.

Prior to treatment initiation, prognostic markers were recorded, including ECOG performance status (0 – 4) and baseline laboratory parameters: serum baseline laboratory parameters: serum albumin (s-Alb, 35–52 g/L), C-reactive protein (CRP, 0–5 mg/L), leukocytes (WBC, 3.8 – 10 ×10^9^/L), platelets (PLT, 140 – 440 ×10^9^/L), absolute neutrophils counts (ANC, 2 – 7 ×10^9^/L), absolute monocytes counts (AMC, 0.2 – 0.8 × 10^9^/L), and absolute lymphocytes counts (ALC, 1.0 – 3.7 × 10^9^/L). These values were used to calculate nutritional and inflammatory biomarkers. All biochemical and hematologic parameters were measured in accredited hospital laboratories, which undergo regular internal and external quality control procedures. The same reference ranges for the monitored laboratory parameters were used across all centers, and all patients had complete data available for all parameters included in the statistical analysis.

PNI calculated as 5 × ALC + s-Alb, with cut-off values of 40.5 and 45. GPS scores (0, 1, 2), CRP (< 10 or > 10 mg/L), NLR (ANC/ALC) with cut-offs of 2.5, 3 and 5, SIRI (ANC × AMC/ALC) with a cut-off of 2.3, and SII (ANC × PLT/ALC) with a cut-off of 900.

Chemotherapy regimens included FOLFOX/CAPOX (5-fluorouracil-folinic acid- oxaliplatin/capecitabine-oxaliplatin), nal-IRI (nanoliposomal irinotecan with 5-FU), and capecitabine. Median OS (mOS) and PFS (mPFS) were evaluated and correlated with prognostic marker values. Adverse events, including hematologic, gastrointestinal and neurologic toxicities, were also recorded.

OS was defined as the interval from the date of the third-line first treatment to the date of a related event, such as death or loss to follow-up. PFS was defined as the interval from the date of the third-line first treatment to death or disease progression. For patients who could not be followed up, the end date was the date of the last known survival. Follow-up was calculated from diagnosis of pancreatic cancer, as well as from beginning of 3rd line treatment.

The primary statistical method used was the Kaplan-Meier survival analysis, from which median survival times and corresponding 95% confidence intervals were calculated. Additionally, basic descriptive statistics were reported: categorical variables were described using absolute and relative frequencies, and continuous variables were summarized using means and standard deviations. For pairwise comparisons of continuous variables across different treatment lines, we used paired Student’s t-tests. To identify factors independently associated with overall survival and progression-free survival, we performed multivariable Cox proportional hazards regression analyses. Data were analysed using IBM SPSS Statistics (Version 20.0, USA). *p* values < 0.05 were considered statistically significant. We presented the therapeutic sequence using a Stankey diagram done with SankeyMatic open source program (sankeymatic.com).

This study was conducted in accordance with the research protocol established by the NieRakovine o.z. study group and approval by the Ethics Review Committee was waived as this is anonymized retrospective study.

## Results

A total of 95 patients received third-line systemic therapy. The mOS for the entire cohort was 5.55 months (range 4.31 – 6.79 months), and the mPFS was 3.29 months (range 2.73 – 3.85 months). Median follow-up from diagnosis was 19.1 months (range: 7.37–135 months), median follow-up from starting 3^rd^ line treatment was 3.2 months (range: 0-28.7 months). The cohort included 47 women and 48 men. The mOS for men was 4.8 months, and for women 6.34 months (95% CI: 4.308 – 6.792, *p* = 0.212, n.s.). The mPFS for men was 3.45 months, and for women 3.02 months (95% CI: 2.733 – 3.847, *p* = 0.855, n.s.). A summary of patient characteristics is presented in **Table 1**.

**Table 1:** Patient characteristics.

Characteristics	Variable	No (%)
Gender	male	48 (50,53%)
	female	47 (49,47%)
Stage od disease	mPDAC (stage IV)	95 (100%)
ECOG PS	0	21 (22,11%)
	1	44 (46,32%)
	2	20 (21,05%)
	3	7 (7,37%)
First line chemotherapy	Gemcitabine-nab-paclitaxel	64 (67,37%)
	FOLFIRINOX	22 (23,16%)
	gemcitabine	6 (6,32%)
	gemcitabine-cisplatin	2 (2,11%)
Second line chemotherapy	nal-IRI	69 (72,63%)
	Gemcitabine-nab-paclitaxel	13 (13,68%)
	gemcitabine	7 (7,3%)
	capecitabine	2 (2,11%)
	FOLFIRINOX	2 (2,11%)
	FOLFOX/CAPOX	2 (2,11%)
Third line chemotherapy	FOLFOX/CAPOX	56 (58,95%)
	nal-IRI	24 (25,26%)
	capecitabine	11 (11,58%)

mPDAC, metastatic pancreatic adenocarcinoma; ECOG PS, performance status according to Easter Cooperative Oncology Group; FOLFOX-5, fluorouracil-folinic acid- oxaliplatin; CAPOX, capecitabine-oxaliplatin; nal-IRI, nanoliposomal irinotecan; FOLFIRINOX-5, fluorouracil-folinic acid-oxaliplatin-irinotecan.

According to performance status, the cohort included 21 patients with ECOG 0, 44 patients with ECOG 1, 20 patients with ECOG 2, and 7 patients with ECOG 3. There was no significant difference in mPFS or mOS according to physician assigned performance status.

The most commonly used regimen was FOLFOX/CAPOX (56 patients), followed by nal-IRI (24 patients) and capecitabine (11 patients). Numerically, the longest median overall survival was for capecitabine at 10.91 months, then for nal-IRI (5.78 months) and lastly for the CAPOX/FOLFOX regimen (5.52 months, *p* = n.s.). The longest mPFS was 3.94 months for capecitabine, 3.45 months for nal-IRI, and 3.02 months for CAPOX/FOLFOX (p = n.s.). A complete summary of the treatment regimens including first- and second-line therapies is presented in **Table 1** and **Figure 1**.

**Figure 2:**
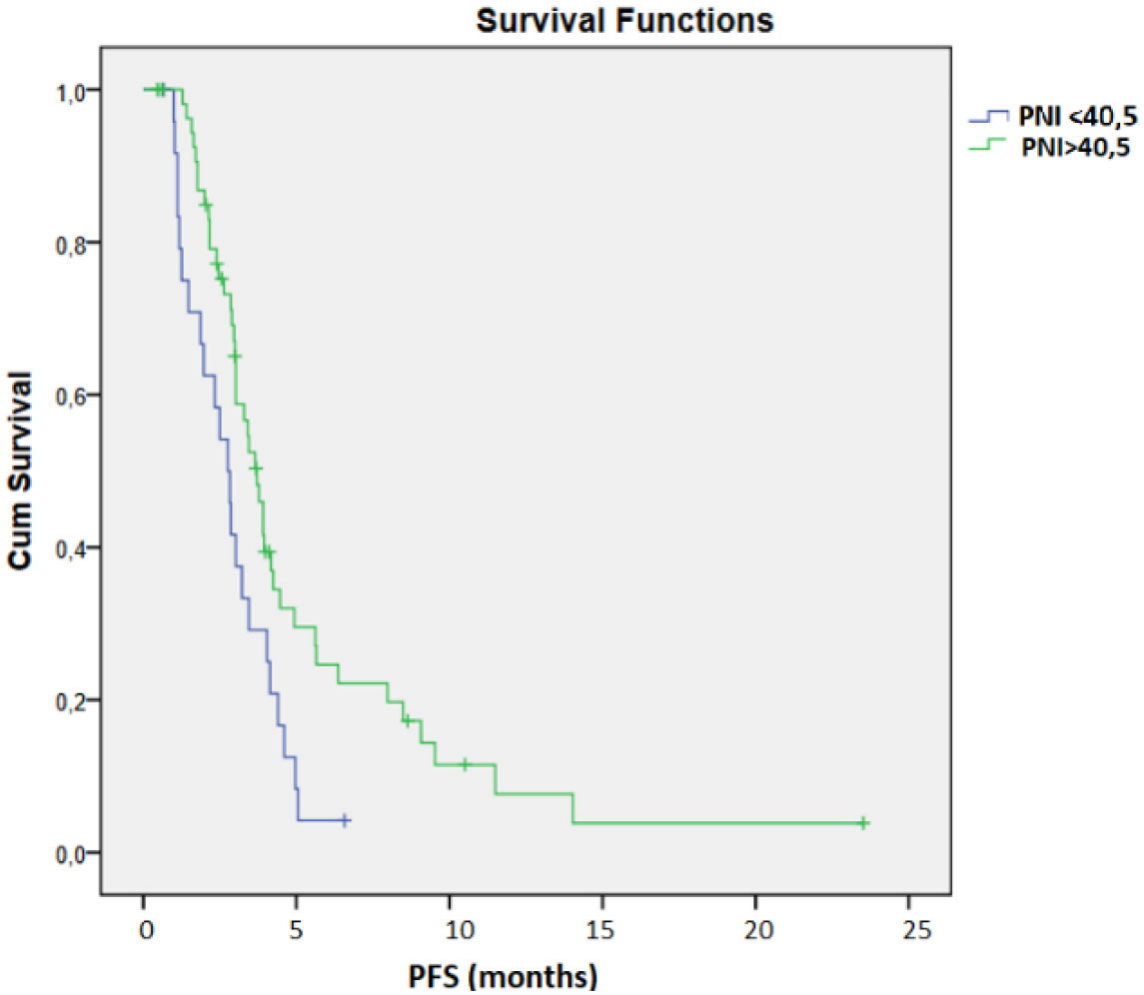
Kaplan–Meier analysis of PFS according to PNI with cut-off 40,5.

**Figure 3:**
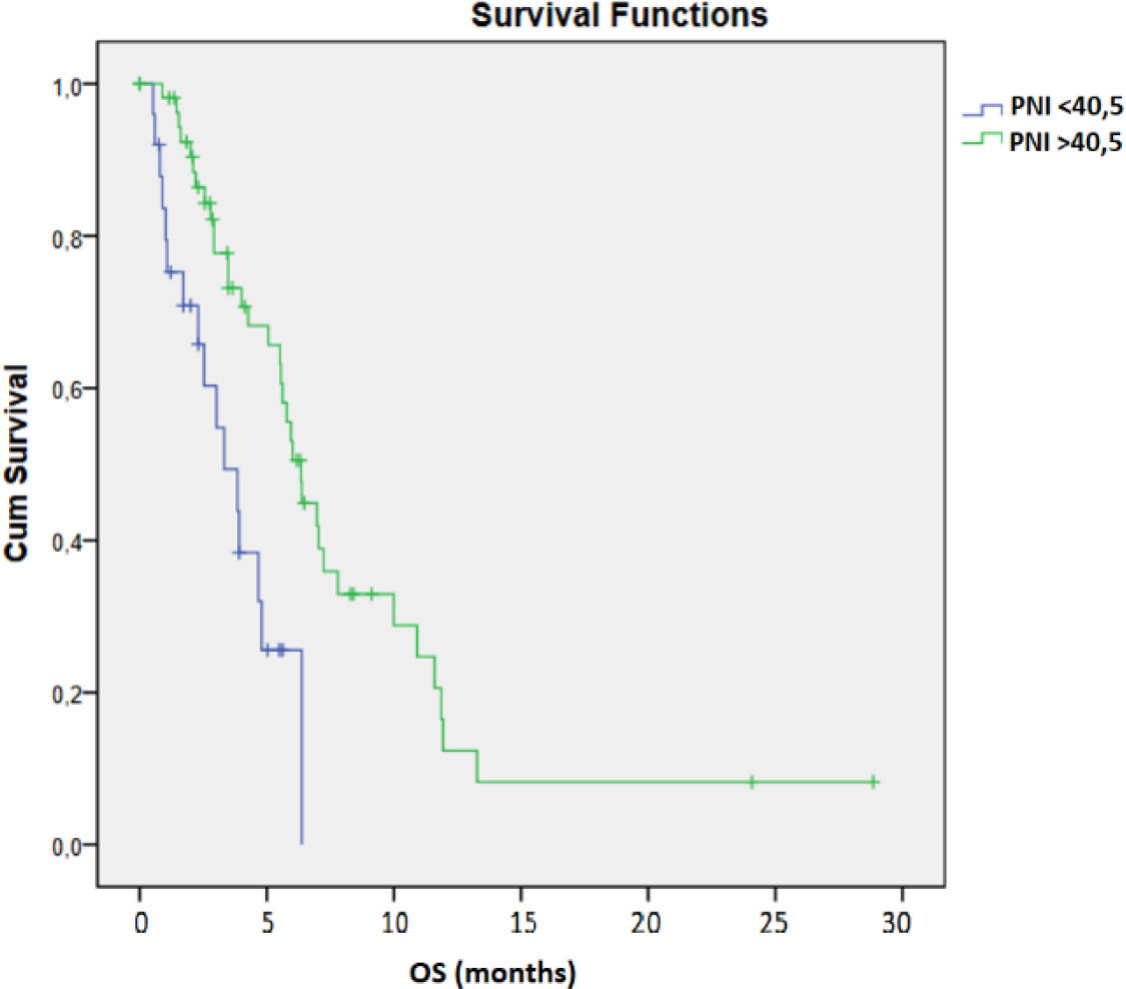
Kaplan–Meier analysis of OS according to PNI with cut-off 40,5.

The mPFS was 2.76 months for patients with PNI < 40.5 and 6.34 months for patients with PNI > 40.5 (95% CI 2.875 – 3.705, *p* = 0.009, **Figure 2**). 25 patients had a PNI < 40.5, while 56 patients had a PNI > 40.5. The mOS for patients with PNI > 40.5 was 6.34 months compared to 3.32 months for those with PNI < 40.5 (95% CI 4.309 – 6.791, *p* = 0.001, **Figure 3**).

38 patients had a PNI > 45, and 43 patients had PNI < 45. The median overall survival for patients with PNI > 45 was 6.34 months compared to 4.27 months for those with PNI < 45 (95% CI 4.309 – 6.791, *p* < 0.051, n.s.). The mPFS was 3.65 months for patients with PNI > 45 versus 3.02 months for those with PNI < 45 (95% CI 2.875 – 3.705, *p* = 0.142).

**Figure 4:**
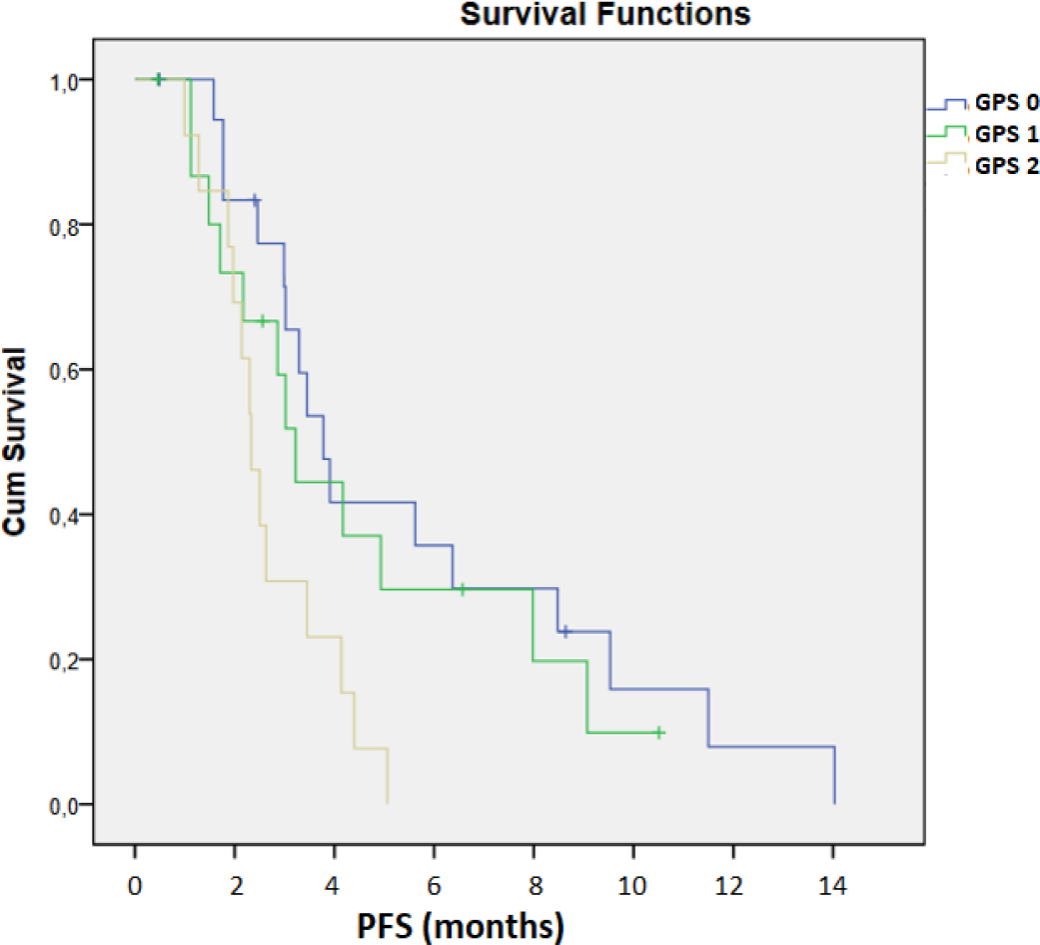
Kaplan–Meier analysis of PFS according to GPS.

**Figure 5:**
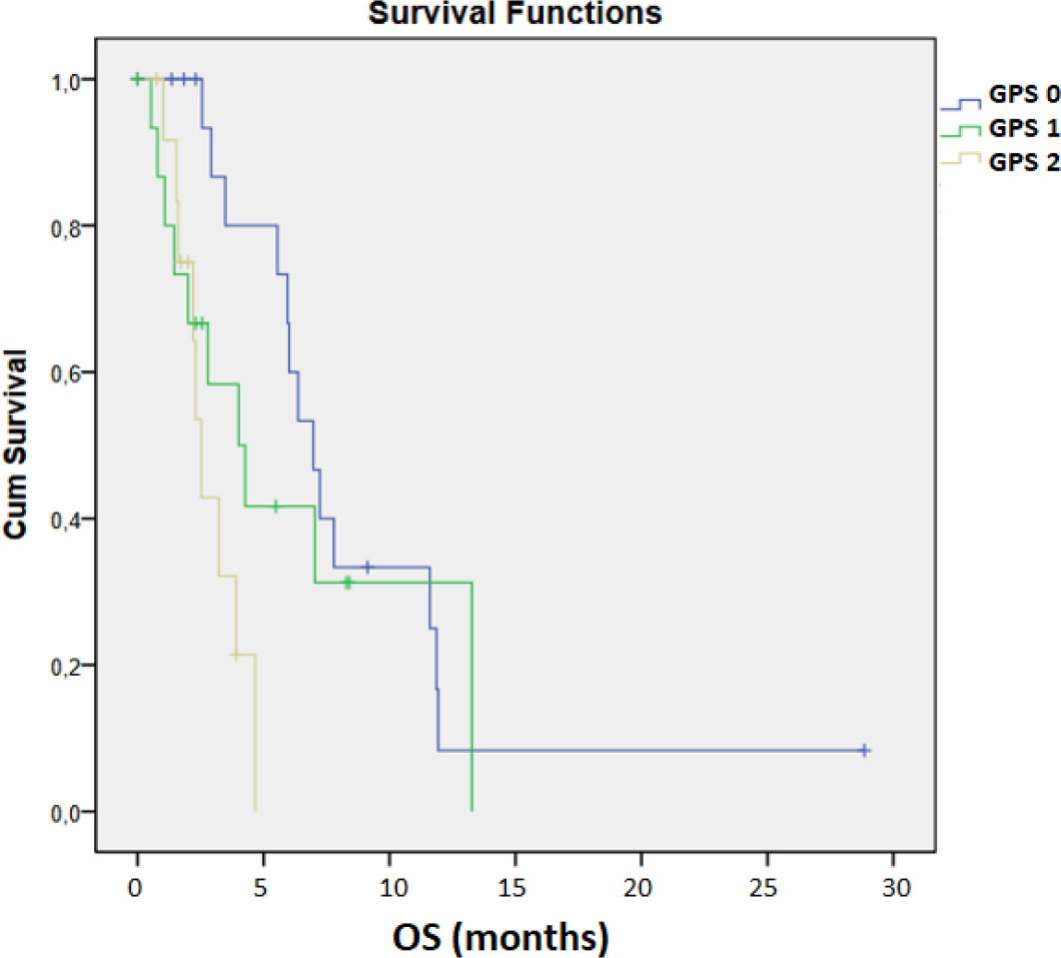
Kaplan–Meier analysis of OS according to GPS.

The mOS in patients with GPS 0 was 6.97 months, 4.01 months in those with GPS 1, and 2.53 months in those with GPS 2 (95% CI: 2.470–6.870, **Figure 4**). The mPFS was 3.78 months for GPS 0, 3.22 months for GPS 1, and 2.33 months for GPS 2 (95% CI 2.736 – 3.704, **Figure 5**). A statistically significant difference between the GPS 0 and GPS 2 groups was confirmed in both analyses for OS and PFS (*p* < 0.001, *p* = 0.008, respectively).

27 patients had CRP < 10 mg/L, and 21 patients had CRP > 10 mg/L. The mOS for patients with CRP < 10 was 6.37 months, compared to 2.79 months for those with CRP > 10 (*p* = n.s.). The mPFS was 3.45 months for CRP < 10 and 2.5 months for CRP > 10 (*p* = n.s.).

40 patients had NLR < 3, and 44 patients had NLR > 3. The mOS for NLR < 3 was 6.01 months versus 5.52 months for NLR > 3 (*p* = n.s.). The mPFS was 3.02 months for NLR < 3 compared to 3.45 months for NLR > 3 (*p* = n.s.).

The number of patients with NLR < 5 was 61, and > 5 was 23. Median overall survival for NLR < 5 was 5.55 months versus 6.37 months for NLR > 5 (*p* = n.s.). The mPFS was 3.02 months for NLR < 5 compared to 3.45 months for NLR > 5 (*p* = n.s.).

The number of patients with NLR < 2.5 was 33, and > 2.5 was 51. The mOS for NLR < 2.5 was 6.01 months versus 5.52 months for NLR > 2.5 (*p* = n.s.). The mPFS was 3.45 months for NLR > 2.5 compared to 3.02 months for NLR < 2.5 (*p* = n.s.),

24 patients had SII > 900, and 60 patients had SII < 900. The mOS for SII > 900 was 4.8 months, compared to 5.62 months for SII < 900 (*p* = n.s.). The mPFS was 3.45 months for SII > 900 versus 3.02 months for SII < 900 (*p* = n.s.).

The number of patients with SIRI < 2.3 was 45, and with SIRI > 2.3 was 39. The mOS for SIRI < 2.3 was 5.62 months compared to 4.27 months for SIRI > 2.3 (*p* = n.s.). The mPFS for SIRI < 2.3 was 3.29 months compared to 3.45 months for SIRI > 2.3 (*p* = n.s.). The complete results are summarized in **Table 2**.

**Table 2:** The results of subgroup analyses.

Markers	No of patients	mOS (months)	95% CI	*p* value	mPFS (months)	95% CI	*p* value
**Gender**			4,308-6,792	0,212		2,733-3,847	0,855
male	48	4,8			3,45		
female	47	6,34			3,02		
**ECOG PS**			4,352-6,888	0,496		2.880-4.020	0,548
0	21	5,52			3,78		
1	44	5,55			3,02		
2	20	6,97			3,71		
3	7	3,91			3,45		
**Chemotherapy regimen**			4,323-6,777	0,792		2,801-3,639	0,396
FOLFOX/CAPOX	56	5,52			3,02		
nal-IRI	24	5,78			3,45		
capecitabine	11	10,91			3,94		
**PNI cut-off 40,5**			4,309-6,791	**0,001**		2,875-3,705	**0,009**
< 40,5	25	3,32			2,76		
> 40,5	56	6,34			3,71		
**PNI cut-off 45**			4,309-6,791	0,051		2,875-3,705	0,142
< 45	43	4,27			3,02		
> 45	38	6,34			3,65		
**GPS**			2,470- 6,870			2,736-3,704	
0	19	6,97			3,78		
1	16	4,01			3,22		
2	13	2,53			2,33		
0 vs 1				0,371			0,480
0 vs 2				**0,001**			**0,008**
1 vs 2				0,164			0,069
**CRP**			2,470-6,870	0,063		2,736-3,704	0,164
< 10mg/l	27	6,37			3,45		
> 10mg/l	21	2,79			2,5		
**NLR cut-off 2,5**			4,308-6,792	0,464		2,748-3,832	0,46
< 2,5	33	6,01			3,02		
> 2,5	51	5,52			3,45		
**NLR cut-off 3**			4,308-6,792	0,176		2,748-3,832	0,81
< 3	40	6,01			3,02		
> 3	44	5,52			3,45		
**NLR cut-off 5**			4,308-6,792	0,993		2,748-3,832	0,793
< 5	61	5,55			3,02		
**>** 5	23	6,37			3,45		
**SIRI**			4,308-6,792	0,085		2,748-3,832	0,620
< 2,3	46	5,62			3,29		
> 2,3	39	4,27			3,45		
**SII**			4,308-6,792	0,482		2,748-3,832	0,681
< 900	60	5,62			3,02		
> 900	24	4,8			3,45		

Values in bold represent statistically significant results.

We evaluated the impact of PS and selected inflammatory biomarkers on PFS. Although statistically significant results were observed in subgroup analyses of PS 3 and in stratifications according to NLR and SIRI, these findings are highly unreliable due to the very small patient numbers and likely reflect random variation rather than true biological effects.

In the multivariate Cox regression analysis, GPS and PNI < 40.5 were identified as independent prognostic factors for OS. Both variables remained statistically significant after adjustment for other covariates included in the model, confirming their independent prognostic value. Consistent results were observed also for PFS.

No statistically significant differences in OS were observed between chemotherapy sequences from first line, including nab-paclitaxel plus gemcitabine → nal-IRI → CAPOX versus other combinations. Median survival from diagnosis of pancreatic cancer was 24.6 months.

Hematologic toxicity (grade 1–3) was observed in 22 patients (23.2%) during third-line therapy, with 20 patients experiencing grade 1 or 2 and 2 patients experiencing grade ≥ 3. Gastrointestinal toxicity (grade 1–2) was observed in 17 patients (17.9%) during third-line therapy. No grade ≥ 3 gastrointestinal toxicities were reported. Neuropathy (grade 1–2) was observed in 3 patients (3.2%) during third-line therapy. No grade ≥ 3 neuropathy events were reported. No significant association was observed between the incidence of toxicity and the studied biomarkers, which may be attributable to the limited sample size. The results are presented in **Table 3**.

**Table 3:** Treatment-related toxicities during third-line therapy.

Toxicity type	Grade	No of patients	% of patients
Hematologic	Any	22	23.3
	1 - 2	20	20.8
	≥ 3	2	2.1
Gastrointestinal	Any	17	17.9
	1 - 2	17	17.9
	≥ 3	0	0
Neuropathy	Any	3	3.2
	1 - 2	3	3.2
	≥ 3	0	0

Patients with a time from pancreatic cancer diagnosis longer than 12 months had significantly longer PFS compared to those with a shorter interval (3.6 vs. 2.4 months, p=0,018). This effect was not observed for overall survival (OS).

## Discussion

This retrospective/prospective observational cohort study (non-randomized) included 95 patients whose data were obtained from SlovPac, the national registry of patients with metastatic pancreatic cancer. We analyzed median overall survival and median progression-free survival and compared these outcomes with nutritional and inflammatory markers. Our cohort achieved a median overall survival of 5.55 months and a median progression-free survival of 3.29 months, which is consistent with findings from recent international studies, shown in **Table 4**.

**Table 4:** Overview of selected studies evaluating treatment regimens and survival outcomes in patients with advanced or metastatic pancreatic cancer in third line.

Study (Author, Year, Country)	No. of patients	mOS (months) (95% CI)	mPFS (months) (95% CI)	Treatment regimens
Lan et al., 2025, Taiwan (3)	257	4.5 (3.6–5.4)	–	Not specified
Gueiderikh et al., 2024, France (25)	251	5.5 (4.8–6.3)	2.03 (1.83–2.36)	Fluoropyrimidine-based regimens, gemcitabine, erlotinib
Kim et al., 2023, South Korea (4)	201	3.5 (2.95- 4.03)	–	Fluoropyrimidine-based regimen, nal-IRI + 5-FU + leucovorin
Lu et al., 2023, China (2)	36	6.9 (0.5–13.9)	4.4 (1.8–7.0)	FOLFIRINOX, FOLFIRI, gemcitabine + nab-paclitaxel, CAPOX, capecitabine, gemcitabine + capecitabine
Dean et al., 2019, Australia (5)	30 (locally advanced)	29.0 (9.0–36.0)	–	Gemcitabine + nab-paclitaxel
Enzler et al., 2024, USA (6)	36	3.7 – 6.3 (1.9–9.3)	1.5-2.4 (0.4–4)	CBP (various doses)/CDDP/nivolumab
Evrard et.al., 2025, France (7)	202	4.2 (2.4- 7.8)	2.2 (1.5-3.9)	FOLFIRI, FOLFOX, 5-FU-cisplatin

CBP, 12-amino-acid calmodulin-binding peptide; cDDP, cisplatin; 5-FU, 5-fluorouracil; CAPOX, capecitabine + oxaliplatin; nal-IRI, nanoliposomal irinotecan; FOLFIRI, folinic acid; 5-FU, irinotecan; FOLFOX, folinic acid; 5-FU, oxaliplatin.

FOLFOX/CAPOX was the most commonly used regimen, followed by nal-IRI and capecitabine. Differences between regimens were not statistically significant, median OS was 10.91 months for capecitabine, 5.78 months for nal-IRI, and 5.52 months for CAPOX/FOLFOX. These results should be interpreted with caution due to the unequal distribution of patients across treatment groups.

Comparison of different treatment sequences from first line, including the most common gemcitabine–nab-paclitaxel → nal-IRI → CAPOX sequence, did not show significant differences in overall survival in our study.

Compared to the cohort study by Evrard et al., 2025 (7), which included 202 patients with unresectable pancreatic cancer receiving third-line chemotherapy, our cohort showed longer mPFS (3.29 vs. 2.2 months) and mOS (5.55 vs. 4.2months). The difference in outcomes may be explained, for example, by the varying number of patients in the cohorts or by the use of different predominant third-line regimens (FOLFOX/CAPOX [our study] vs. FOLFIRI [study by Evrard et al.]). Furthermore, while Evrard et al. analyzed standard laboratory parameters such as albumin, bilirubin, CA 19-9, and NLR, our study focused on nutritional and inflammatory markers, where GPS and PNI demonstrated promising prognostic value.

Patients with PNI > 40.5 achieved significantly better mOS (6.34 vs. 3.32 months, *p* = 0.001). The results are consistent with previous studies highlighting the importance of nutritional status as an independent prognostic factor. Results using a cut-off of 45 also suggested a trend towards improved survival, although differences were not statistically significant.

Inflammatory markers, specifically the GPS and CRP also correlated with survival. The best mOS and mPFS were observed in patients with GPS 0 (mOS 6,97 months), while patients with GPS 2 had the poorest prognosis (mOS 2,53 months, p < 0,006). CRP values < 10 mg/L were associated with a trend toward longer survival, though the difference was not statistically significant (mOS 6.37 vs. 2.79 months, *p* < 0.063). These findings support the notion that CRP, as a marker with a 10 mg/L cut-off value, may have prognostic relevance even in third-line treatment of mPDAC.

In contrast, leukocyte-based markers such as NLR, SIRI and SII did not show statistically significant associations with OS or PFS. Nonetheless, consistent trends were observed: lower NLR and SII values correlated with better numerical survival, suggesting potential clinical utility in larger cohorts or prospective studies. The lack of statistical significance may be attributed to cohort heterogeneity and limited subgroup sizes.

There were no significant differences in survival between sexes or across ECOG performance status groups. This may reflect a selection bias where only patients with relatively preserved performance status were considered fit for third-line treatment, resulting in a more homogeneous population in terms of functional status. Performance status was crucial for treatment tolerability in these patients, as therapy often needs to be stopped due to clinical deterioration or rising CA 19-9. This highlights the importance of using biomarkers before treatment to identify patients no longer suitable for therapy.

Treatment toxicity was acceptable, with most adverse events being mild to moderate in severity. The incidence of severe hematologic toxicity (grade 3) was low, and neurotoxicity occurred in only a minority of patients. Confirming a potential association between higher treatment-related toxicity and nutritional or inflammatory biomarkers would require substantially larger and more complete datasets than those available in the present study.

Limitations of this study include its retrospective cohort-based design, limited sample size, and potential inconsistencies in the assessment of toxicity and performance status. The non-randomized selection of third-line regimens may have influenced the findings. Additional studies are needed to validate and expand on these observations. The goal is to establish standardized cut-off values for biomarkers and define their practical application in clinical practice. Future prospective studies should incorporate predefined time points for biomarker assessment (e.g. CRP, nutritional parameters as well as ctDNA) at baseline and during treatment. Collection and analysis of these biomarkers should be standardized and, ideally, centralized in a single reference laboratory to minimize analytical variability.

In routine practice, these biomarkers could complement overall patient assessment and help determine eligibility for systemic therapy. These biomarkers should help select patients fit for chemotherapy and those likely to benefit more from best supportive care. Such decisions are particularly relevant in patients with metastatic PDAC after failure of second-line palliative chemotherapy. Biomarkers could also provide valuable information during clinical progression in third-line therapy, for instance when performance status deteriorates or CA 19–9 rises and imaging assessments are not routinely conducted.

For instance, prospective validation of PNI and GPS in a larger, multi-national cohort would strengthen the evidence base. Additionally, exploring the combination of nutritional and inflammatory biomarkers with other clinical or molecular markers such as DNA (mutations and methylations), RNA, protein biomarkers and circulating tumor cells could help in developing risk stratification for these patients. Moreover, the generalizability of these findings to non-European populations should be considered, as nutritional and inflammatory profiles may vary by ethnicity and socio-economic groups.

## Conclusion

Our study provides evidence that nutritional and inflammatory biomarkers may have prognostic value in patients with metastatic pancreatic cancer undergoing third-line treatment. While numerous studies have focused on the identification of biomarkers for the diagnosis of pancreatic ductal adenocarcinoma; however, only a limited number have investigated their utility in predicting treatment response or their impact on survival outcomes. PNI, GPS, and CRP emerged as strong predictive factors for both OS and PFS, and they may help in identifying patients suitable for further lines of therapy. Integrating genomic and transcriptomic analyses with clinical and nutritional-inflammatory profiles may improve understanding of treatment efficacy and resistance mechanisms. Such information could help guide treatment decisions by identifying patients who are likely to benefit from therapy and those for whom best supportive care is more appropriate.

## Data Availability

The raw data supporting the conclusions of this article will be made available by the authors, without undue reservation.
