# Supplementary material for: Survival outcomes and prognostic value of nutritional and inflammatory markers in third-line treatment of metastatic pancreatic cancer
Source: Front Oncol. 2026 Mar 24;16:1713376. doi: 10.3389/fonc.2026.1713376 (PMC13054719; doi:10.3389/fonc.2026.1713376)

**Supplementary material: Kaplan-Meier Curves of Nutritional and Inflammatory Markers in mPDAC**


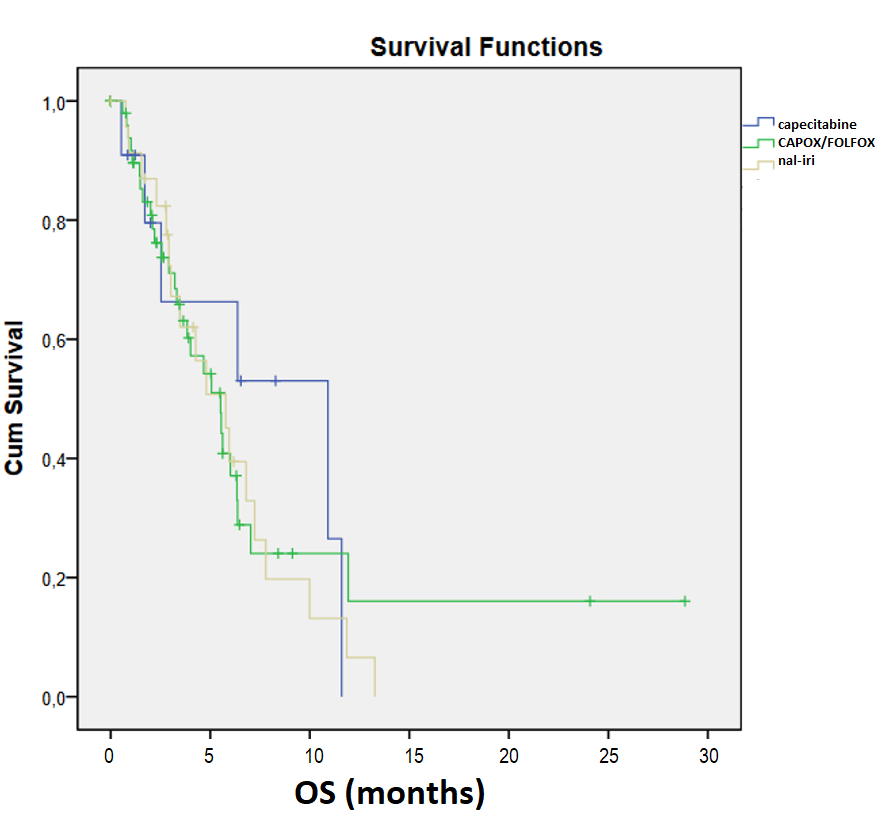

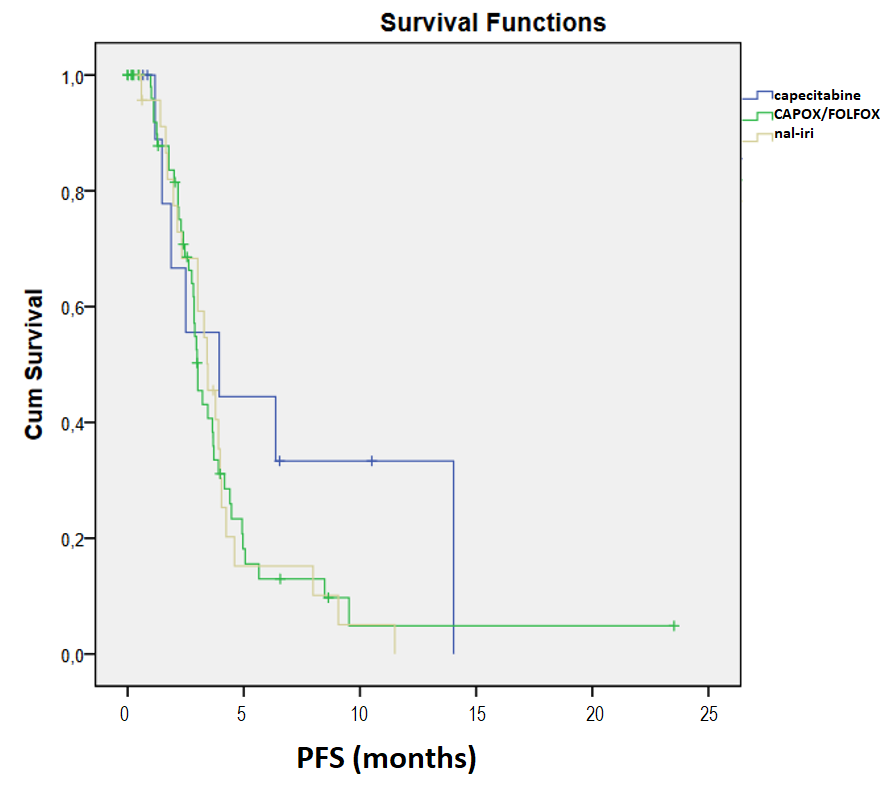


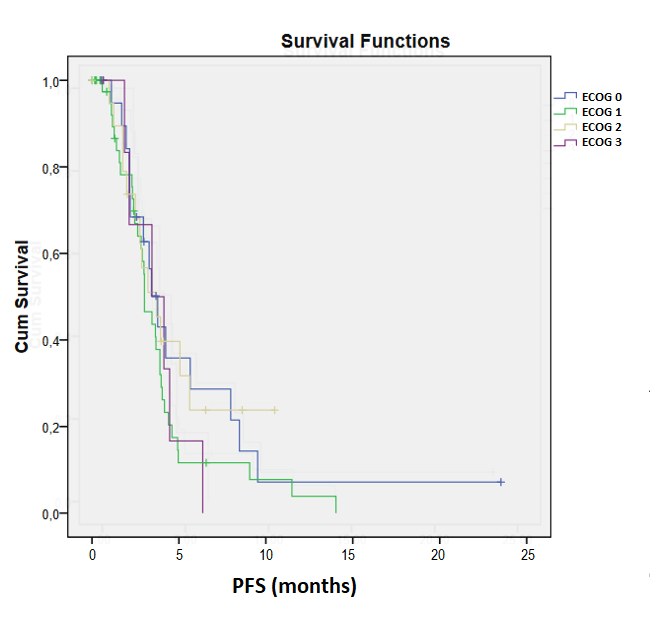

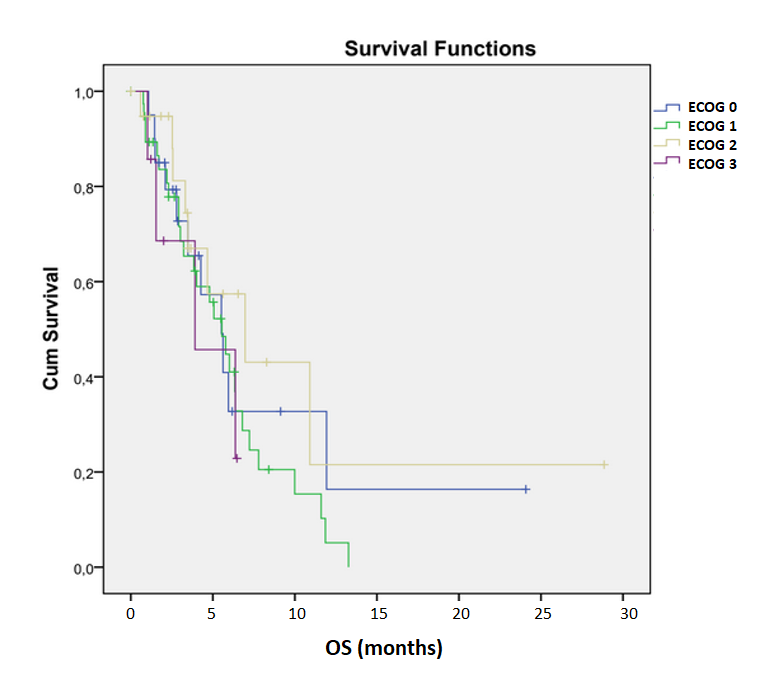


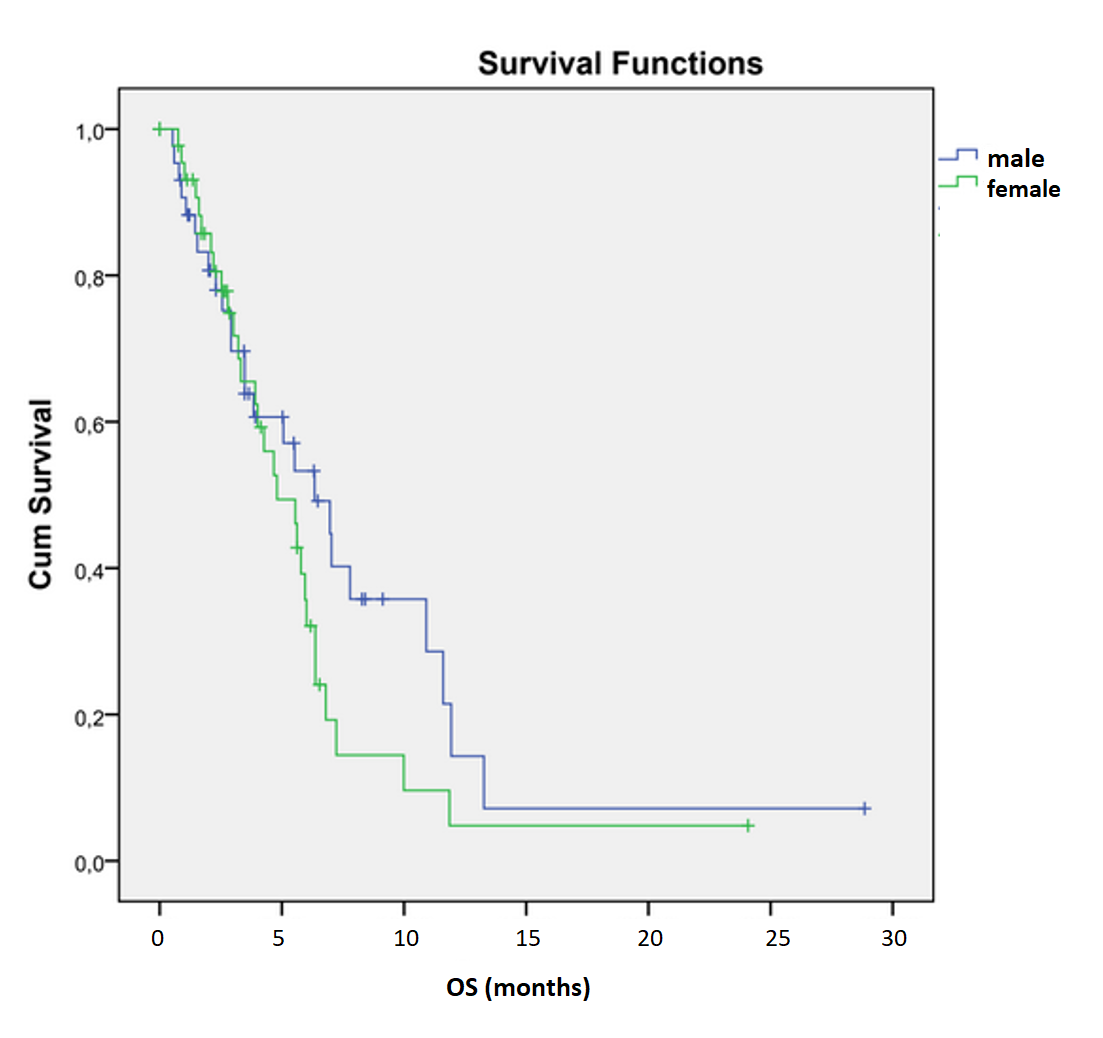

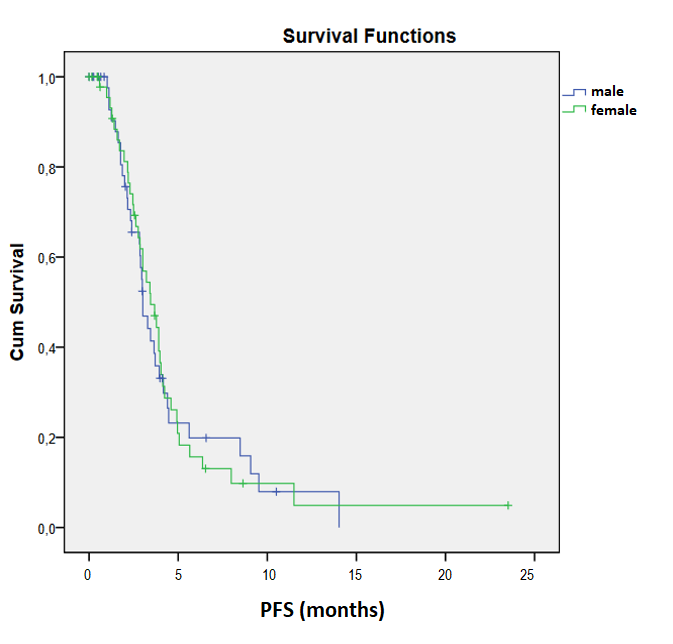


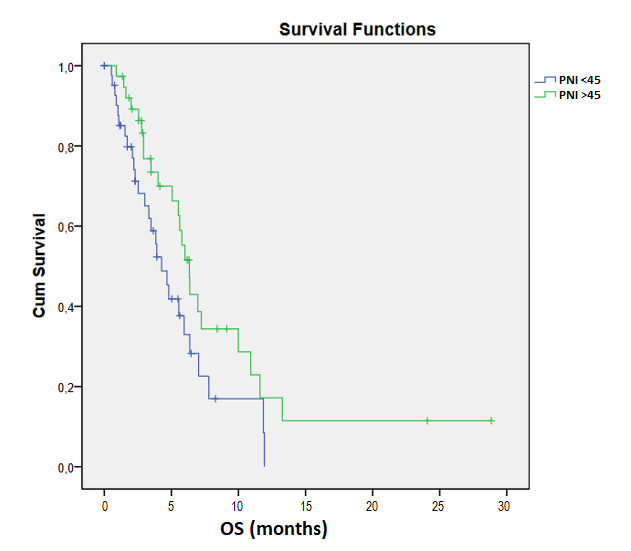

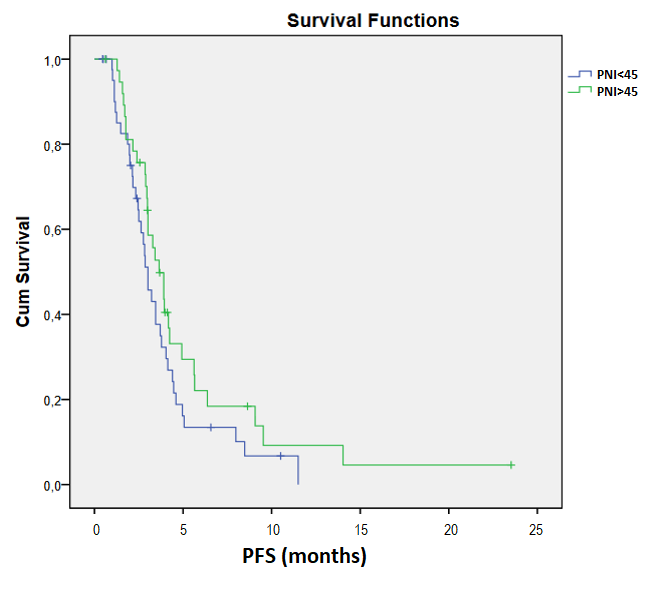


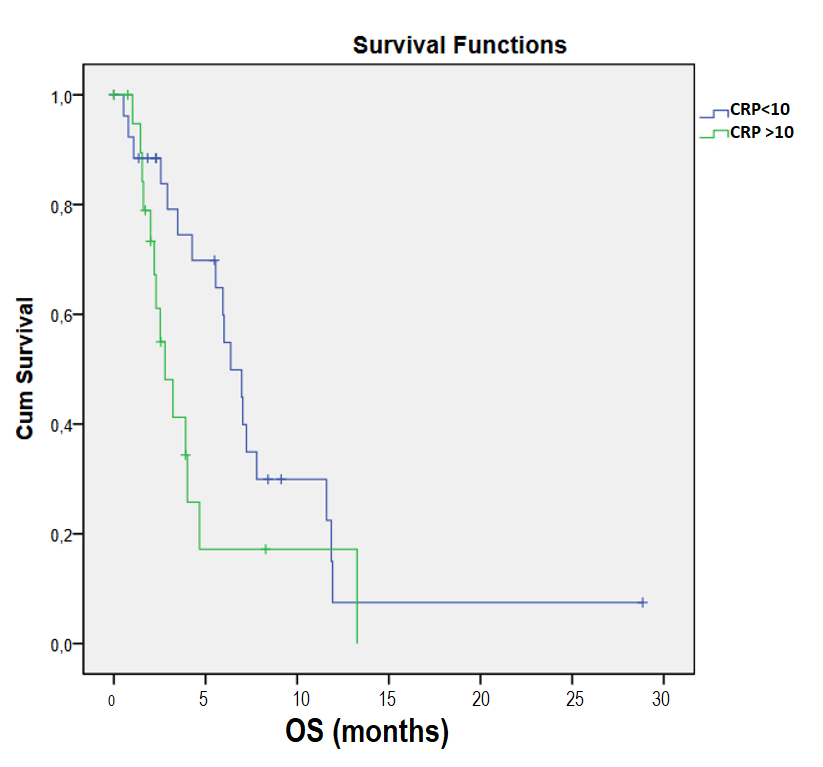

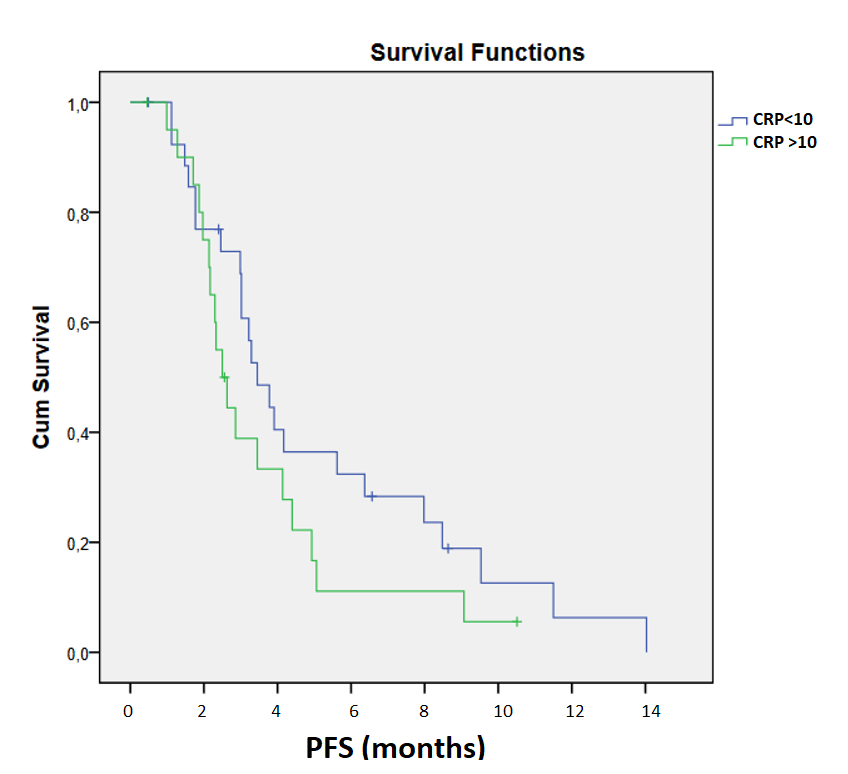


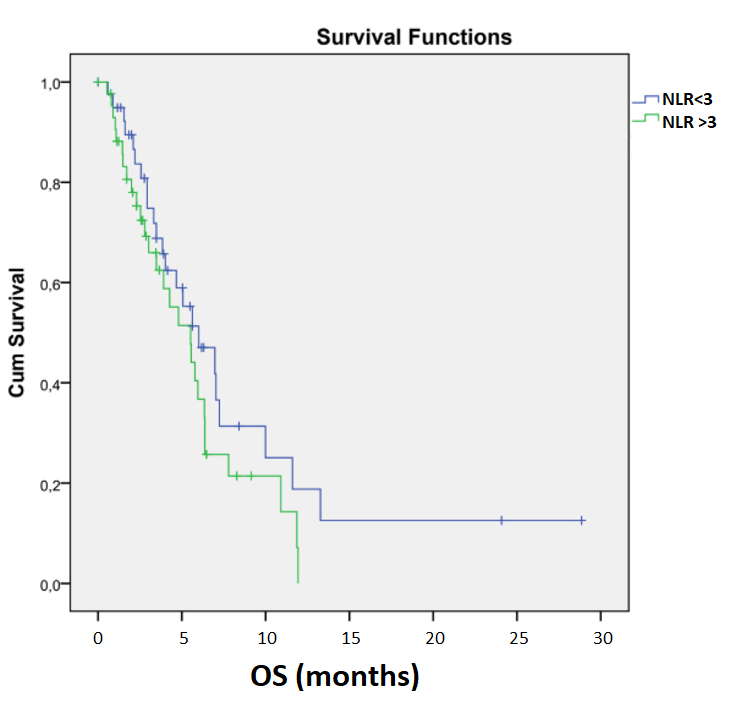

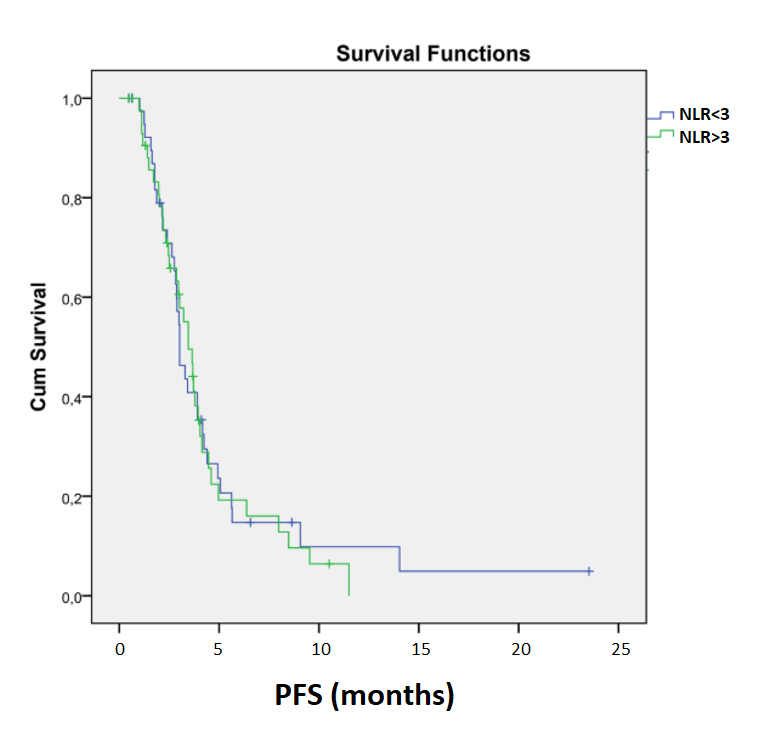


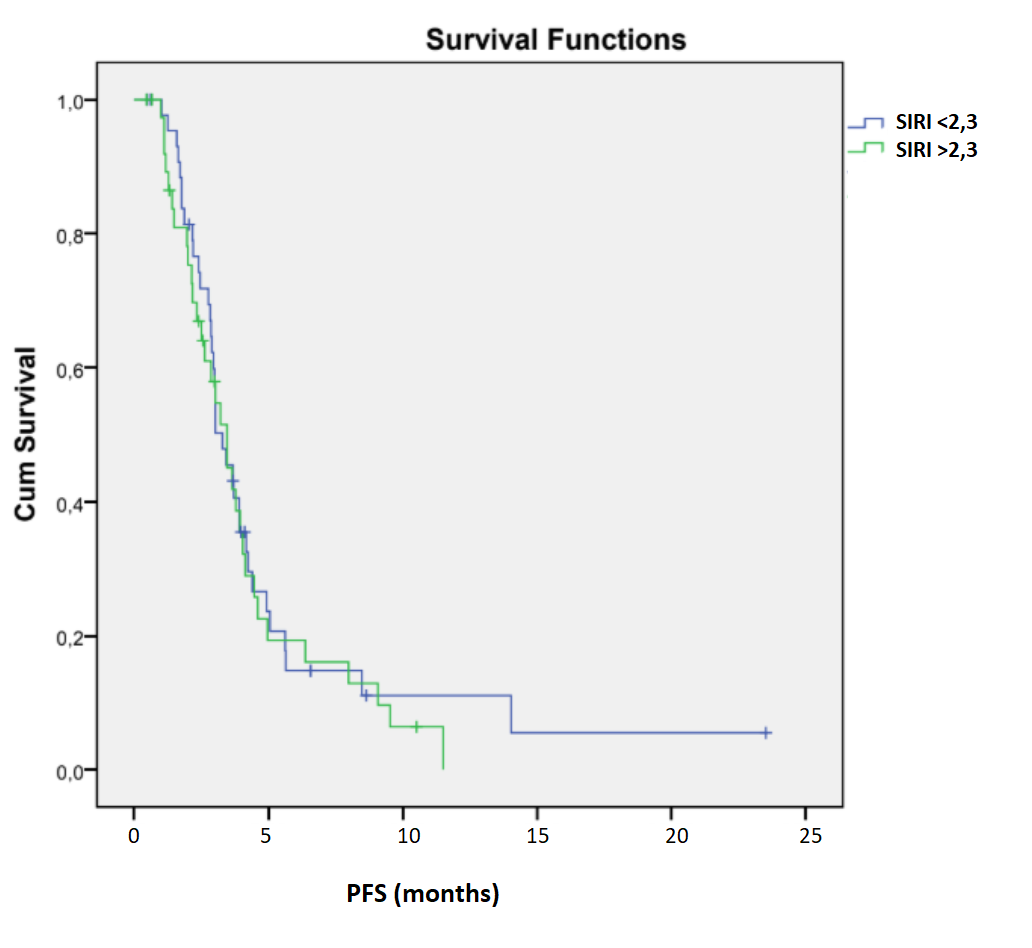

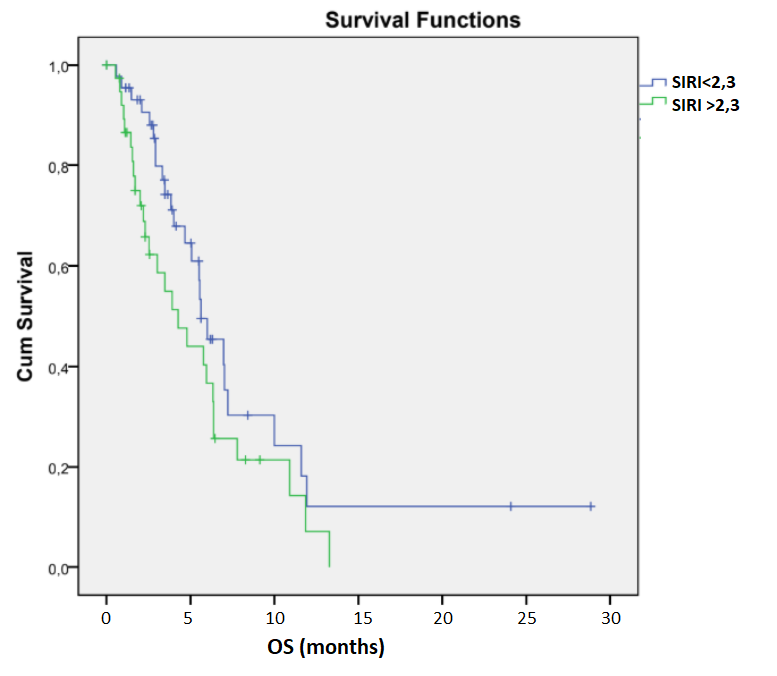


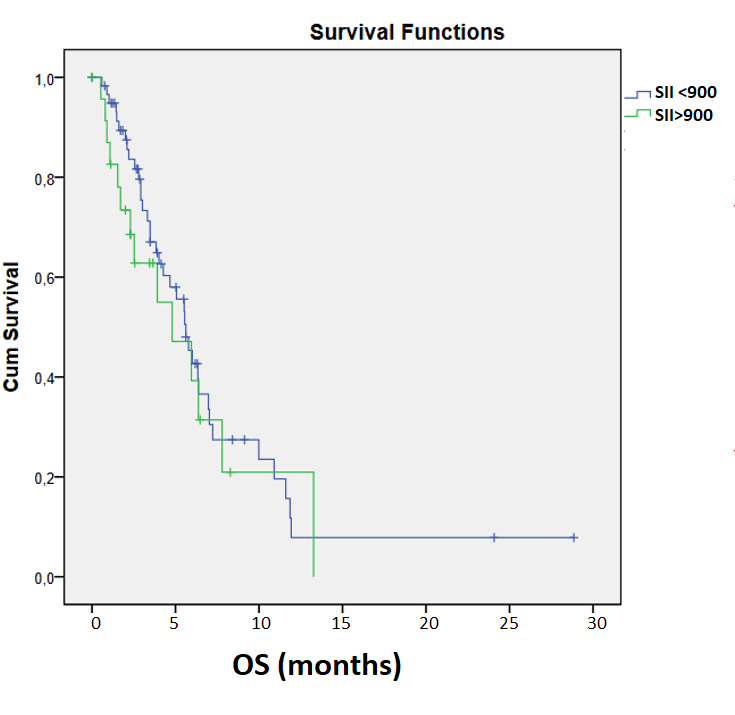

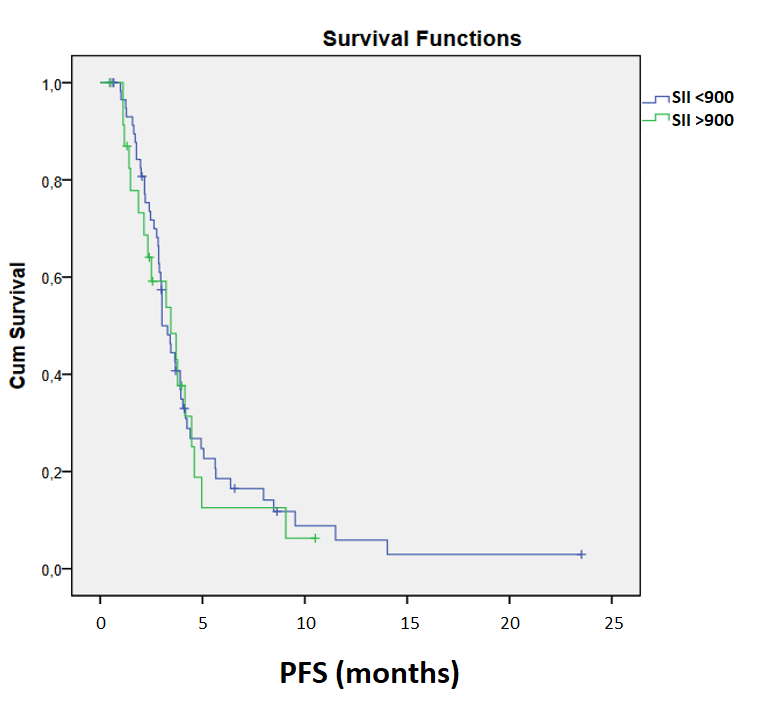


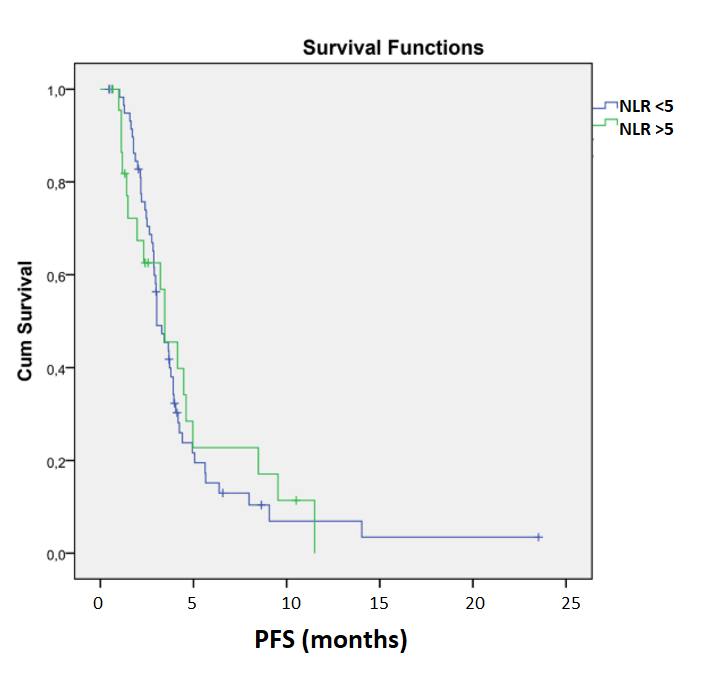

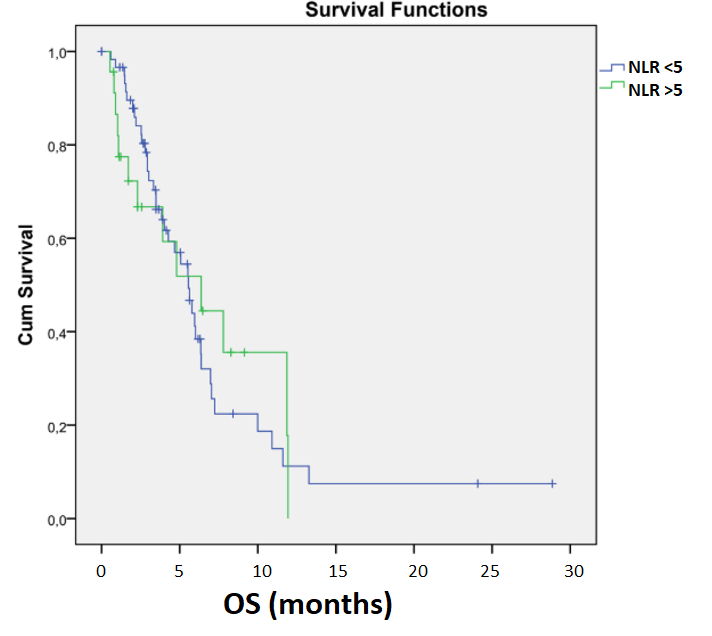


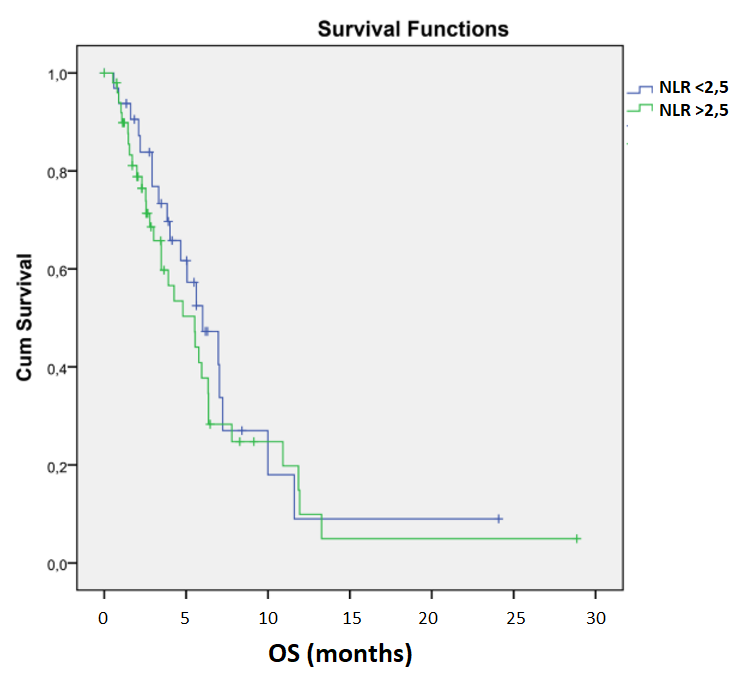

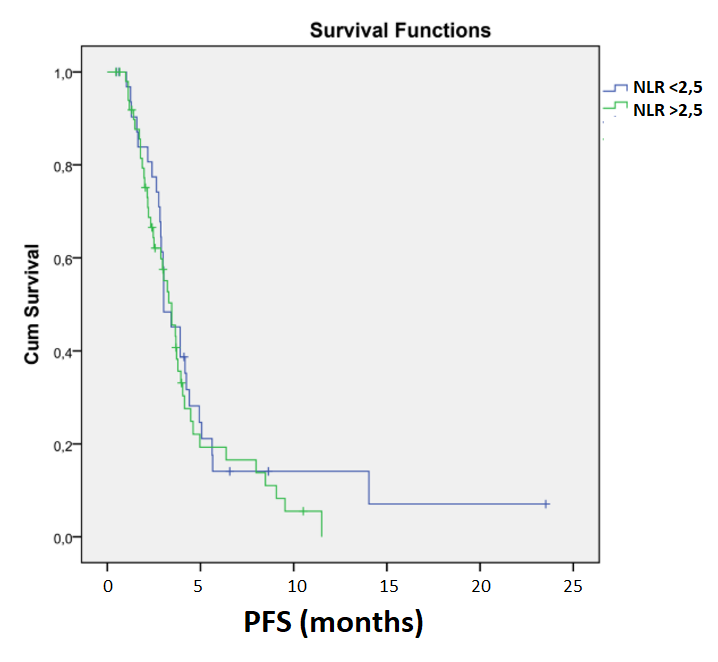

Supplement: Supplementary file 1 [file DataSheet1.docx]
